# A Novel Germline Mutation of KEAP1 (R483H) Associated with a Non-Toxic Multinodular Goiter

**DOI:** 10.3389/fendo.2016.00131

**Published:** 2016-09-20

**Authors:** Eijun Nishihara, Akira Hishinuma, Takahiko Kogai, Nami Takada, Mitsuyoshi Hirokawa, Shuji Fukata, Mitsuru Ito, Tomonori Yabuta, Mitsushige Nishikawa, Hirotoshi Nakamura, Nobuyuki Amino, Akira Miyauchi

**Affiliations:** ^1^Center for Excellence in Thyroid Care, Kuma Hospital, Kobe, Japan; ^2^Department of Infection Control and Clinical Laboratory Medicine, Dokkyo Medical University, Mibu, Japan

**Keywords:** KEAP1 germline mutation, multinodular goiter, familial goiter, NRF2 expression, Graves’ disease

## Abstract

**Background:**

A germline mutation of *KEAP1* gene was reported as a novel genetic abnormality associated with familial multinodular goiter. That report was limited, and the pathogenic features were not well established.

**Patient findings:**

We report a 47-year-old Japanese woman who presented with hyperthyroidism and a large multinodular goiter. The family history was notable for a paternal history of goiter. Graves’ disease was diagnosed based on positive TRAb, but scintiscan imaging showed that the patient’s radioiodine uptake was restricted in the non-nodular areas, indicating largely cold nodules. A total thyroidectomy was performed. The resected thyroid tissue weighed 209 g, and subsequent pathological findings were benign. The patient had a germline heterozygous KEAP1 mutation, c. 1448 G > A, resulting in an amino acid substitution (p.R483H). A next-generation sequencing analysis covering all known genes associated with multinodular goiter showed no additional germline mutation. The nuclear accumulation of NRF2, a protein associated with KEAP1, was shown at much higher rates in the patient’s nodules compared with nodules obtained from four unrelated patients with multinodular goiters.

**Conclusion:**

A novel germline mutation (R483H) of *KEAP1* gene was associated with the development of a non-toxic multinodular goiter.

## Introduction

The development of a multinodular goiter is associated with various factors such as genetic abnormalities, iodine deficiency, and natural goitrogens. A germline mutation of Kelch-like ECH-associated protein 1 (*KEAP1*) gene was reported as a novel molecular cause of familial multinodular goiter (1). This mutation is a frameshift mutant (D294T, fs*23) in KEAP1, and the truncated KEAP1 protein is not generated (1). KEAP1 was originally identified as a protein associated with nuclear factor erythroid-2-related factor 2 (NRF2) (2) and also functions as a substrate adaptor protein for a Cul3-dependent E3 ubiquitin ligase complex, with a subsequent degradation of NRF2 by the proteasome (3). Oxidative stress or electrophiles inactivate KEAP1 by the modification of cysteine residues in KEAP1, which results in the decline of ubiquitin activity and thereby the facilitation of the nuclear accumulation of NRF2 shifting from the cytoplasmic localization. As a key molecule for the response of oxidative stresses or electrophiles, NRF2 activates the transcription of various cytoprotective genes that enhance cell proliferation (4).

In another aspect, KEAP1 mutations are sufficient to lead to a constitutive activation of NRF2 by disrupting the KEAP1–NRF2 interaction. Somatic mutations in *KEAP1* gene in cancer tissues and cancer-derived cell lines of different origins affect the repressive activity of KEAP1, stimulate the nuclear accumulation of NRF2, and provide advantages for cell growth (4). However, a germline mutation in *KEAP1* has not yet been reported in familial cancer cases; it was detected only in a family with multinodular goiter (1). In this report, we describe a hyperthyroid patient who had a large multinodular goiter. She had a family history of goiter and carried a germline mutation (R483H) in *KEAP1* gene.

## Patient

### The Patient and Her Family

**Figure 1:**
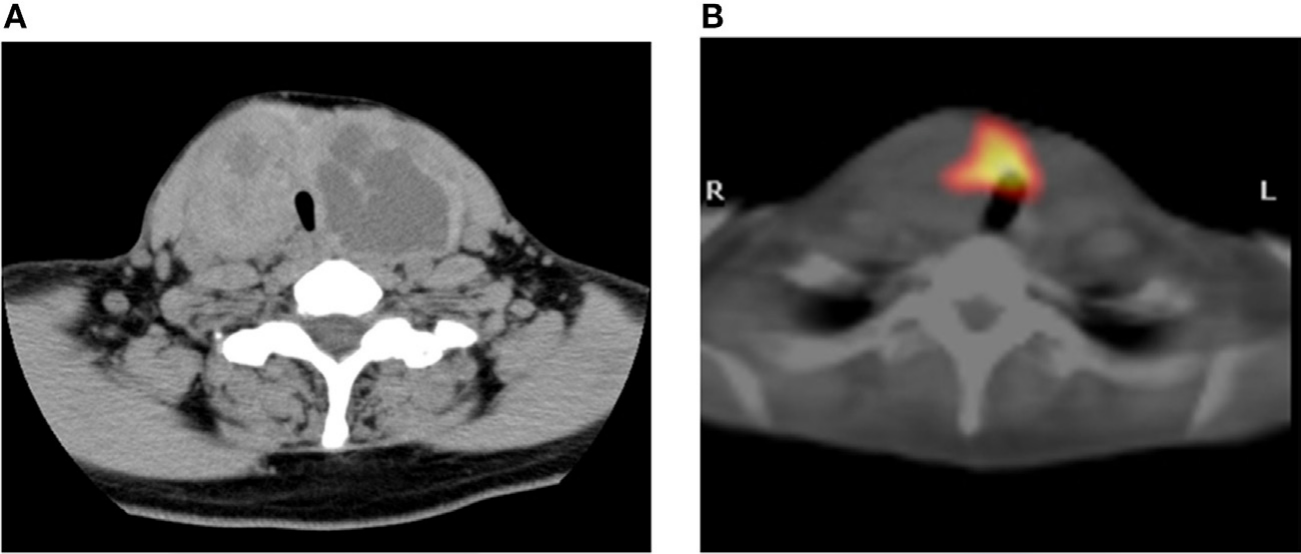
**Imaging study**. CT scan showing multinodular enlargement of the thyroid gland **(A)**. The corresponding region of radioiodine uptake represents cold nodules in this SPECT/CT fusion image **(B)**.

A 47-year-old Japanese woman consulted our hospital for the examination of a large goiter. Neck computed tomography (CT) showed multinodular lesions in both lobes (Figure 1A), and there were no abnormal findings on chest CT. She presented with subclinical hyperthyroidism (FT4: 1.53 ng/dl, FT3: 3.47 pg/ml, and TSH: 0.011 μIU/ml) and high levels of thyroglobulin (Tg; 742.5 ng/ml). Anti-thyroid peroxidase (TPO) antibodies and anti-Tg antibodies were negative. Scintiscan imaging showed radioiodine uptake in the non-nodular area of the thyroid isthmus, but absence in the nodular lesions of both lobes (Figure 1B). The radioiodine uptake at 3 h was 8.3% within the normal range.

Specimens obtained from the thyroid nodules by fine-needle aspiration were categorized as benign based on the Bethesda system. Seven months later, the diagnosis of Graves’ disease was made based on the patient’s overt hyperthyroidism (FT4: 3.17 ng/dl, FT3: 12.27 pg/ml, and TSH: <0.003 μIU/ml) and positive TRAb (7.9 IU/l). She entered remission 25 months after starting methimazole therapy, but the Graves’ hyperthyroidism relapsed thereafter. She underwent a total thyroidectomy that included the persistently large goiter, and the weight of the resected thyroid tissue was 209 g.

## Materials and Methods

### Screening of Goiter-Associated Gene Variants by Next-Generation Sequencing

We performed a next-generation sequencing (NGS) analysis targeted to most of the coding exons of goiter-associated genes (Goiter Ampliseq panel, Table 1), using custom primers designed by Ion Ampliseq Designer (Life Technologies, Carlsbad, CA, USA), according to the manufacturer’s instructions. Briefly, genomic DNA was isolated from whole blood samples with a QIAamp DNA Blood Mini Kit (Qiagen, Hilden, Germany), and a multiplex polymerase chain reaction (PCR) of a total of 429 amplicons (Table 1) was performed, followed by the addition of Ion Xpress Barcode Adaptors, with the Ion Ampliseq Library Kit 2.0 (Life Technologies).

**Table 1:** **Genes and exons included in Goiter Ampliseq panel**.

Gene	Chromosomes	No. of exons	Incomplete exons
*NRAS*	chr1	4	
*TPO*	chr2	18	Exons 8 and 9
*THRB*	chr3	8	
*IYD*	chr6	7	
*SLC26A4*	chr7	21	Exon 17
*TG*	chr8	48	
*RET*	chr10	21	Exon 1
*HRAS*	chr11	5	
*KRAS*	chr12	5	
*TSHR*	chr14	12	
*DICER1*	chr14	27	
*DUOX2*	chr15	34	Exons 5, 6, and 7
*DUOXA2*	chr15	6	
*SLC5A5*	chr19	15	Exons 1, 4, 7, and 12
*KEAP1*	chr19	5	

After enrichment by clonal emulsion PCR on Ion Sphere particles (Ion PGM Template OT2 200 Kit), the barcoded libraries were loaded on an Ion 318 chip, and massively paralleled sequencing was carried out on an Ion Torrent PGM sequencer with the Ion PGM Sequencing 200 Kit version 2.

### Sequence Data Analysis

We analyzed the raw signal data of NGS by using Torrent Suite software (ver. 5.0.4), including adaptor trimming, read alignment to human genome 19 reference, coverage analysis, and variant calling. To determine whether the detected sequence variants are known pathogenic mutations or novel variants, we carried out variant filtration as well as annotation by using Ion Reporter software (ver. 5.0; Life Technologies). Sequence variant confirmation was performed by conventional Sanger methods with a BigDye Terminator Cycle Sequencing Kit (Applied Biosystems, Foster City, CA, USA). This study was approved by the Ethics Committee of Kuma Hospital, and informed consent was obtained from the patient and her family members for the use of their blood and tissue samples for research purposes.

### Histopathological Evaluation and Immunohistochemistry

After surgical resection, the thyroid tissues were routinely fixed in 10% neutral buffered formalin, and specimens were embedded in paraffin. Serial sections (3-μm thick) were cut from each paraffin block. For the light-microscopic examination, the sections were stained with hematoxylin–eosin (HE). The immunostaining for human NRF2 (rabbit polyclonal, C-20, 1:50, Santa Cruz Biotechnology, Santa Cruz, CA, USA) was performed using the Leica Bondmax system (Leica Microsystems, Wetzlar, Germany) and a Bond refine kit (Leica Microsystems) according to the manufacturer’s instructions. Antigen retrieval was performed using Bond Epitope Retrieval Solution 1 (pH 6) at 100°C for 60 min. Control specimens were obtained from four unrelated patients with large multinodular goiters (>100 g) and negative anti-TPO and anti-Tg antibodies.

## Results

### Histopathology and Family History

**Figure 2:**
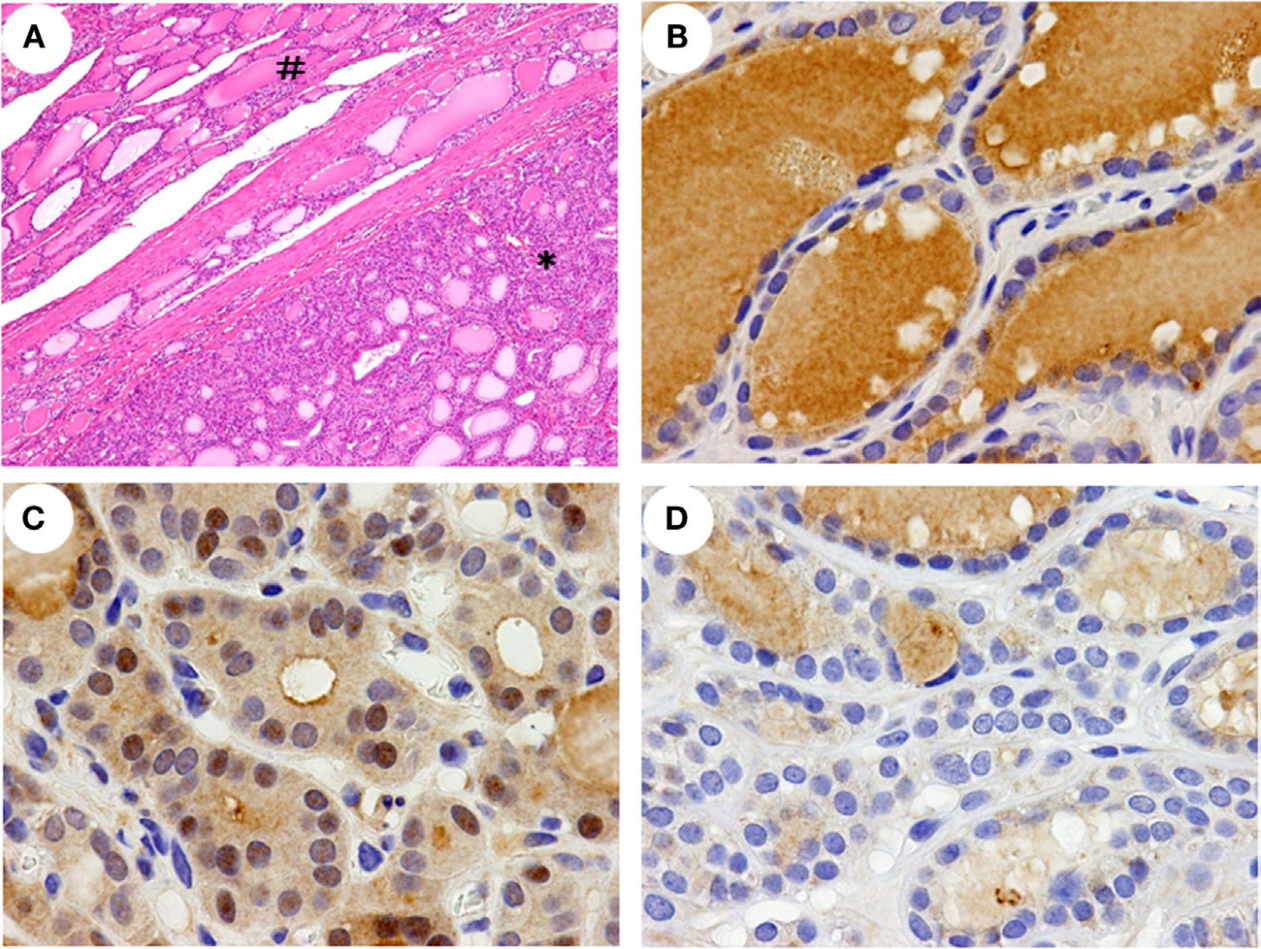
**Histopathological features and immunohistochemical detection of NRF2 in the thyroid**. A histopathological feature **(A)** of a left lobe shows non-nodular parenchyma (#) and a partially encapsulated follicular nodule (*). A greater nuclear accumulation of NRF2 was detected in a nodular lesion of the patient **(C)** than in a non-nodular parenchyma of the patient **(B)** and a nodular lesion in a control **(D)**. The data shown are representative of two experiments with similar results. Original magnification; 40× (**A**), 400× **(B–D)**.

The subsequent histopathological findings of the thyroid specimens showed that both lobes of the thyroid was almost completely occupied by multinodular lesions that were partially encapsulated by fibrous tissue and concomitantly degenerative cystic structures without lymphoid infiltration in the stroma. The nodules were composed of various-sized thyroid follicles and showed largely high cellularity due to a microfollicular pattern with columnar epithelial cells (Figure 2A). The internodular thyroid parenchyma showed almost normal morphology, and malignant findings were not identified.

**Figure 3:**
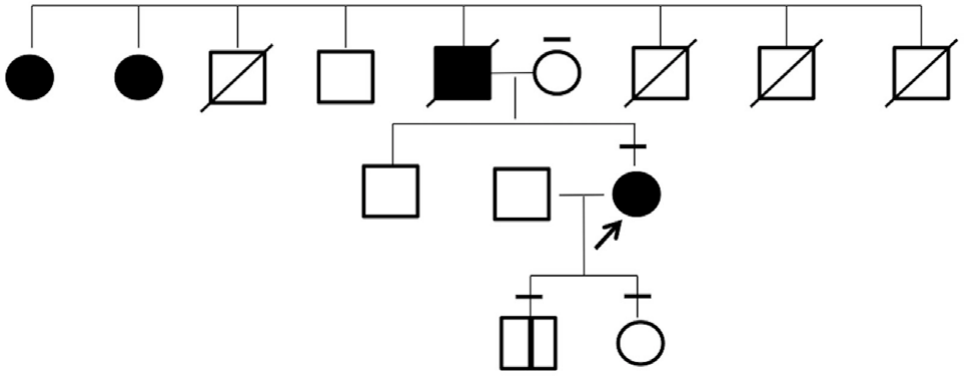
**Pedigree of the investigated family**. Individuals affected with goiter are indicated by filled symbols. The arrow shows the proband in this study. The bars above each symbol indicate individuals who underwent a sequencing analysis for *KEAP1* gene.

The patient’s father had presented with a marked goiter but had died earlier due to head injury. She had a family history of large goiter in two paternal aunts who were unavailable to undergo genetic testing (Figure 3). The patient’s mother, son, and daughter were euthyroid and had no goiter or nodular lesion on ultrasonography of the neck.

### Identification of KEAP1 Gene Mutation

To identify any known germline mutation of goiter-associated genes, we first performed a targeted-NGS analysis of the patient’s genomic DNA from peripheral blood leukocytes with the Goiter Ampliseq panel (Table 1). The proband had 27 exonic variants identified in the Single Nucleotide Polymorphism database (dbSNP) and one heterozygous exonic variant in *KEAP1* (c.1448 G > A, p.R483H) not registered in the dbSNP. According to the NCBI’s ClinVar database,^1^ the detected 27 variants in dbSNP, including 16 missense polymorphisms, have not been reported as pathogenic mutations.

**Figure 4:**
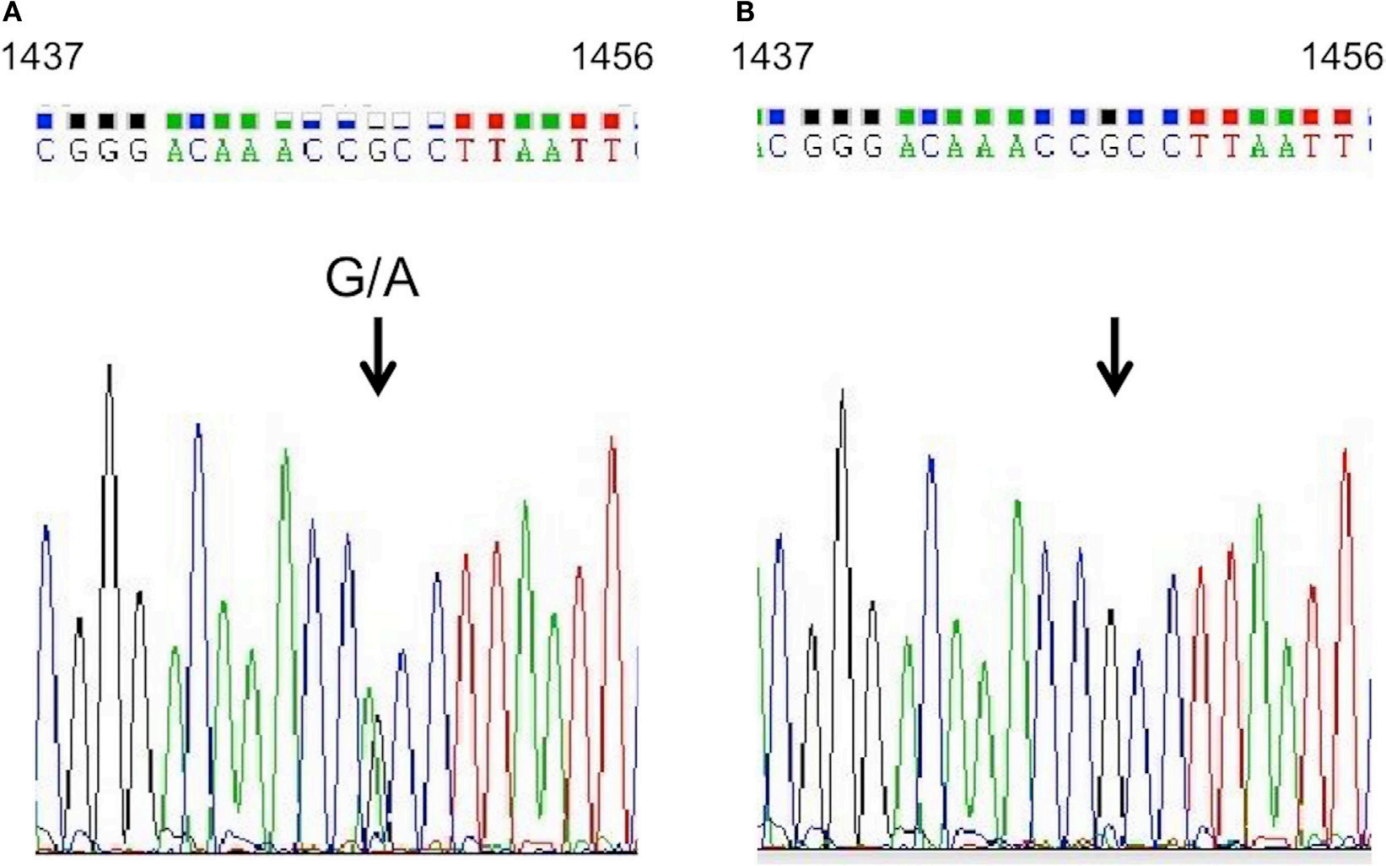
***KEAP1* gene analysis**. Sequencing analysis of *KEAP1* in genomic DNA extracted from the proband’s peripheral blood leukocytes. A heterozygous guanine-to-adenine transition at position 1448 (indicated by arrows) was identified in the proband **(A)**, and a wild-type sequence was shown in her mother **(B)**.

Although several exons of target genes were not completely sequenced with the Goiter Ampliseq panel (Table 1), conventional Sanger sequencing indicated no pathogenic polymorphism in those exons. Our subsequent analysis by Sanger methods validated the heterozygous mutation of c.1448 G > A in *KEAP1* in the patient (Figure 4A).

Our sequence analysis of genomic DNA from the proband’s 16-year-old son showed an identical heterozygous guanine-to-adenine transition at position 1448; however, it was absent in the proband’s daughter and mother (Figure 4B). The R483H variant was evaluated as “disease causing or deleterious” by Mutant Taster,^2^ PolyPhen2,^3^ PROVEAN,^4^ and PANTHER.^5^ This variant was not described previously in other individuals by the 1000 Genome Project^6^ or the Exome Aggregation Consortium.^7^

### NFR2 Expression in the Thyroid

In this patient, NRF2 positive staining in the nuclei was detected in >30% of the cells in nodular lesions (Figure 2C) but in <10% of the cells in the non-nodular parenchyma (Figure 2B). In all four control specimens, a nuclear accumulation of NRF2 was detected in <10% of the cells in both the nodular lesions and the non-nodular parenchyma (Figure 2D and data not shown).

## Discussion

We report a hyperthyroid patient harboring a novel germline mutation (R483H) in *KEAP1* gene who presented with a large multinodular goiter and Graves’ disease. The R483H mutation in *KEAP1* is not present as a polymorphism in several databases. The amino acid residue of R483 is strongly conserved in mammals and is essential for the molecular interaction with NRF2 (5). The binding capability of a mutant KEAP1 with NRF2 can be estimated by the cellular localization of NRF2, which presents with nuclear accumulation by a dissociation of KEAP1 binding in the cytoplasm (2). Indeed, the nuclear accumulation of NRF2 was shown at much higher rates in the nodules in this patient than in the nodules obtained from four unrelated patients with multinodular goiter (Figures 2B–D).

The direct target genes of NRF2 include the synthesis and conjugation of glutathione, antioxidant substance, and metabolic enzymes, which can protect from oxidative stresses that may occur during thyroid hormone synthesis and confer multiple advantages of cell proliferation (4). In Keap1–null mice, these target genes of NRF2 are constitutively upregulated, whereas its upregulation is reversed in Keap1/Nrf2 double-deficient mice (6), suggesting that impaired KEAP1 function leads to the constitutive stabilization of NRF2. Although a previous report showed that the lower expression of wild-type KEAP1 by a frameshift mutation is a possible pathogenesis of multinodular goiter (1), our present analysis is the first to demonstrate that a single amino acid substitution in *KEAP1* could lead to the development of a multinodular goiter.

Somatic mutations in *KEAP1* were reported to occur in 60% of lung cancers (7). The R483H somatic mutation in *KEAP1* has been reported in lung cancer tissue with elevated levels of NRF2 expression (8). In addition, oncogenes, such as *KRAS, BRAF*, and *C-MYC*, can stimulate NRF2 transcription and activation in cancer cells (9). Concomitant abnormalities of oncogenes and *KEAP1* gene may affect tumorigenesis and accelerate the growth of cancer cells. On the other hand, the presence of the R483H germline mutation in *KEAP1* did not result in any nuclear accumulation of NRF2 in the non-nodular parenchyma of our patient, and no thyroid nodule was detected by ultrasonography in her 16-year-old son, who has same *KEAP1* mutation as the patient. Our NGS analysis, which covered all known genes associated with multinodular goiter including a battery of thyroid hormone synthesis genes, *TSH receptor*, and *DICER1* showed no additional genetic abnormalities in the patient. These findings suggest that the nodular formation in multinodular goiter may be influenced by microenvironmental changes including loss of heterozygosity of *KEAP1*, because the thyroid gland is most susceptible to oxidative stress during hormone synthesis, which can lead to an additional somatic mutation at a high frequency.

It is unknown whether this *KEAP1* mutation may affect any aspect of Graves’ disease, but the thyroid gland of our patient was almost completely occupied by cold nodules without lymphoid infiltration in the stroma, suggesting that her large goiter was due not to her Graves’ disease but rather to the cold nodules. Collectively, our findings indicated that a novel germline mutation (R483H) of *KEAP1* gene is associated with the development of a non-toxic multinodular goiter as one pathogenesis.

## Author Contributions

Study design: EN. Experiment: EN, AH, TK, and NT. Interpretation of data: EN, AH, TK, NT, MH, SF, MI, TY, MN, HN, NA, and AM. Drafting the work or revising it critically: EN, AH, TK, NT, MH, SF, MI, TY, MN, HN, NA, and AM. Final approval of the version to be published: EN, AH, TK, NT, MH, SF, MI, TY, MN, HN, NA, and AM. Agreement to be accountable for all aspects of the work: EN, AH, TK, NT, MH, SF, MI, TY, MN, HN, NA, and AM.

## Conflict of Interest Statement

The authors declare that the research was conducted in the absence of any commercial or financial relationships that could be construed as a potential conflict of interest.

## Footnotes

^1^http://www.ncbi.nlm.nih.gov/clinvar/

^2^http://www.mutationtaster.org/

^3^http://genetics.bwh.harvard.edu/pph2/index.shtml

^4^http://provean.jcvi.org/seq_submit.php

^5^http://www.pantherdb.org/tools/csnpScoreForm.jsp

^6^http://www.1000genomes.org/

^7^http://exac.broadinstitute.org/

## References

[1] Teshiba R Tajiri T Sumitomo K Masumoto K Taguchi T Yamamoto K. Identification of a KEAP1 germline mutation in a family with multinodular goitre. PLoS One (2013) 8:e65141. 10.1371/journal.pone.0065141 23724128 PMC3665763

[2] Itoh K Wakabayashi N Katoh Y Ishii T Igarashi K Engel JD Keap1 represses nuclear activation of antioxidant responsive elements by Nrf2 through binding to the amino-terminal Neh2 domain. Genes Dev (1999) 13:76–86. 10.1101/gad.13.1.76 9887101 PMC316370

[3] Kobayashi A Kang MI Okawa H Ohtsuji M Zenke Y Chiba T Oxidative stress sensor Keap1 functions as an adaptor for Cul3-based E3 ligase to regulate proteasomal degradation of Nrf2. Mol Cell Biol (2004) 24:7130–9. 10.1128/MCB.24.16.7130-7139.2004 15282312 PMC479737

[4] Suzuki T Motohashi H Yamamoto M. Toward clinical application of the Keap1-Nrf2 pathway. Trends Pharmacol Sci (2013) 34:340–6. 10.1016/j.tips.2013.04.005 23664668

[5] Tong KI Katoh Y Kusunoki H Itoh K Tanaka T Yamamoto M. Keap1 recruits Neh2 through binding to ETGE and DLG motifs: characterization of the two-site molecular recognition model. Mol Cell Biol (2006) 26:2887–900. 10.1128/MCB.26.8.2887-2900.2006 16581765 PMC1446969

[6] Wakabayashi N Itoh K Wakabayashi J Motohashi H Noda S Takahashi S Keap1-null mutation leads to postnatal lethality due to constitutive Nrf2 activation. Nat Genet (2003) 35:238–45. 10.1038/ng1248 14517554

[7] Li QK Singh A Biswal S Askin F Gabrielson E. KEAP1 gene mutations and NRF2 activation are common in pulmonary papillary adenocarcinoma. J Hum Genet (2011) 56:230–4. 10.1038/jhg.2010.172 21248763 PMC3268659

[8] Takahashi T Sonobe M Menju T Nakayama E Mino N Iwakiri S Mutations in Keap1 are a potential prognostic factor in resected non-small cell lung cancer. J Surg Oncol (2010) 101:500–6. 10.1002/jso.21520 20213688

[9] DeNicola GM Karreth FA Humpton TJ Gopinathan A Wei C Frese K Oncogene-induced Nrf2 transcription promotes ROS detoxification and tumorigenesis. Nature (2011) 475:106–9. 10.1038/nature10189 21734707 PMC3404470

